# Sleep Duration and Functional Disability Among Chinese Older Adults: Cross-Sectional Study

**DOI:** 10.2196/53548

**Published:** 2024-06-10

**Authors:** Minjing Luo, Yue Dong, Bingbing Fan, Xinyue Zhang, Hao Liu, Changhao Liang, Hongguo Rong, Yutong Fei

**Affiliations:** 1 Center for Evidence-Based Chinese Medicine Beijing University of Chinese Medicine Beijing China; 2 Beijing GRADE Centre Beijing China; 3 School of Traditional Chinese Medicine Beijing University of Chinese Medicine Beijing China; 4 Dongzhimen Hospital Affiliated to Beijing University of Chinese Medicine Beijing China; 5 Institute for Excellence in Evidence-Based Chinese Medicine Beijing University of Chinese Medicine Beijing China

**Keywords:** sleep duration, functional disability, activity of daily living disability, instrumental activity of daily living, older population

## Abstract

**Background:**

The duration of sleep plays a crucial role in the development of physiological functions that impact health. However, little is known about the associations between sleep duration and functional disability among older adults in China.

**Objective:**

This study aimed to explore the associations between sleep duration and functional disabilities in the older population (aged≥65 years) in China.

**Methods:**

The data for this cross-sectional study were gathered from respondents 65 years and older who participated in the 2018 survey of the China Health and Retirement Longitudinal Study, an ongoing nationwide longitudinal investigation of Chinese adults. The duration of sleep per night was obtained through face-to-face interviews. Functional disability was assessed according to activities of daily living (ADL) and instrumental activities of daily living (IADL) scales. The association between sleep duration and functional disability was assessed by multivariable generalized linear models. A restricted cubic-spline model was used to explore the dose-response relationship between sleep duration and functional disability.

**Results:**

In total, 5519 participants (n=2471, 44.77% men) were included in this study with a mean age of 73.67 years, including 2800 (50.73%) respondents with a functional disability, 1978 (35.83%) with ADL disability, and 2299 (41.66%) with IADL disability. After adjusting for potential confounders, the older adults reporting shorter (≤4, 5, or 6 hours) or longer (8, 9, or ≥10 hours) sleep durations per night exhibited a notably increased risk of functional disability compared to that of respondents who reported having 7 hours of sleep per night (all *P*<.05), which revealed a U-shaped association between sleep duration and dysfunction. When the sleep duration fell below 7 hours, increased sleep duration was associated with a significantly lower risk of functional disability (odds ratio [OR] 0.85, 95% CI 0.79-0.91; *P*<.001). When the sleep duration exceeded 7 hours, the risk of functional disability associated with a prolonged sleep duration increased (OR 1.16, 95% CI 1.05-1.29; *P*<.001).

**Conclusions:**

Sleep durations shorter and longer than 7 hours were associated with a higher risk of functional disability among Chinese adults 65 years and older. Future studies are needed to explore intervention strategies for improving sleep duration with a particular focus on functional disability.

## Introduction

The issue of disability in the older population has garnered significant attention and interest in recent decades [1-3]. Disability—as defined by the International Classification of Functioning, Disability, and Health—encompasses the combined effects of impairments, activity limitations, or participation restrictions [4]. Functional disability is a significant measure of restrictions in activities, specifically referring to challenges in performing basic activities of daily living (ADLs) and/or instrumental activities of daily living (IADLs) [5,6]. ADLs are widely recognized as essential tasks for maintaining independence in one’s own residence, such as dressing, bathing, and eating. IADLs refer to more complex tasks that require a higher level of independence and cognitive ability, such as managing financial matters, engaging in shopping activities, and preparing meals [7].

According to the World Health Organization (WHO) survey of 2022, 46.1% of adults 60 years and older worldwide are living with a disability, and this figure is increasing in tandem with the rapid global aging phenomenon [8]. China has one of the highest proportions of older citizens globally, with over 14% of individuals living in China classified as older people (ie, 65 years and older) [8]. This proportion is expected to increase to 30% by 2050, along with a massive drop in the elderly support ratio (defined as the number of people of “working age” [15-64 years] divided by those aged ≥65 years) from 9 in 2010 to 3 in 2050, which is comparable to that of the United States and Germany [9]. A meta-analysis conducted in 2022 reported that the cumulative prevalence of functional disability in China exceeds 30 million [10]. Moreover, the WHO projects that by 2050, the number of older adults living with a functional disability in China will increase to 66 million [11]. Given the high and rising prevalence of functional disabilities among the growing older adult population, exploring the key factors influencing the risk of functional disabilities is crucial to establish appropriate prevention and intervention strategies.

Duration of sleep plays a crucial role in the development of physiological functions that impact health, showing correlations with an increased risk of cognitive decline, depression, cardiovascular diseases, osteoporosis, and stroke [12-17]. However, a consensus has yet to be reached about the association between the duration of sleep and potential risk of functional disability. 

In 2016, the National Survey of Midlife Development in the United States presented evidence that insufficient sleep is an independent and important factor contributing to physiological function disability [18]. A cohort study conducted in China with 1798 individuals 90 years or older demonstrated that sleep duration of 8 to 10 hours was associated with the lowest risk of experiencing an ADL disability, whereas a sleep duration exceeding 12 hours was associated with a heightened risk of experiencing ADL disability [19]. However, there is limited knowledge about the correlations of sleep duration with IADL disability. A US study with 136 participants, predominantly comprising older (aged≥65 years) Black individuals from low-income households, revealed a significant correlation between extended sleep duration surpassing 7.5 hours and worse IADL performance [20]. Similarly, evidence from the National Health Interview Survey spanning 2000 to 2015 showed that an extended sleep duration (≥9 hours) was associated with a higher risk of IADL disability [21]. In addition, owing to interactions with historical, ethnic, economic, and sociocultural factors, the association between sleep duration and functional disability may be heterogeneous across different countries and regions [22,23]. Therefore, the aim of this study was to examine the association between sleep duration and functional disability in China using a nationally representative sample of adults 65 years and older.

## Methods

### Design and Study Population

This study used data from the China Health and Retirement Longitudinal Study (CHARLS), a comprehensive longitudinal data set designed to provide a representative sample of individuals aged ≥45 years residing in mainland China [3]. The baseline survey of the CHARLS involved a multistage, stratified, probability proportional to sampling method to recruit participants across 150 counties or districts and 450 villages or urban areas throughout the country. Face-to-face interviews were used to obtain the data. A relative or caregiver was asked to complete the survey on behalf of the older adult if assistance was needed. Additional details regarding the CHARLS data set are available elsewhere [3].

**Figure 1:**
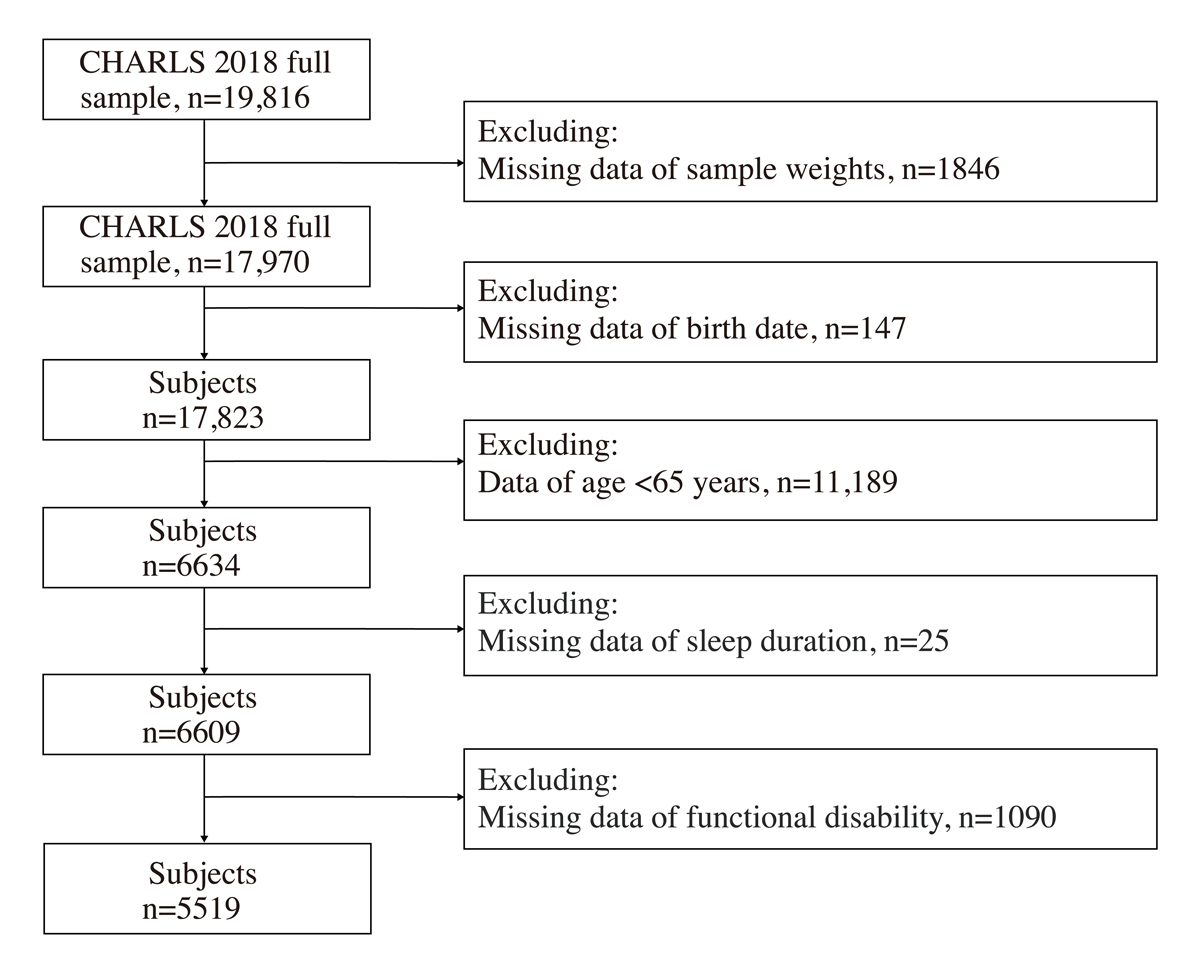
Flowchart of the study sample inclusion process. CHARLS: China Health and Retirement Longitudinal Study.

The data analyzed in this study were taken from the most recent wave of the CHARLS in 2018, with a sample size of 19,816 participants, to investigate the potential correlation between sleep duration and functional disability. We included only observations without missing values from Chinese older adults aged ≥65 years. The schematic flow of the study sample is depicted in Figure 1. The total sample consisted of 5519 individuals, both with and without functional disability.

This cross-sectional study followed the STROBE (Strengthening the Reporting of Observational Studies in Epidemiology) reporting guideline [24].

### Ethical Considerations

The protocols followed in the CHARLS were aligned with the principles of the Declaration of Helsinki [25]. This study obtained ethical approval from the Institutional Review Board at Peking University (IRB00001052-11015, IRB00001052-11014) [3]. All participants in the CHARLS provided written informed consent. The participants received a physical examination report for their participation. The data were deidentified and treated for confidentiality by anonymization and coding.

### Sleep Duration

The self-reported data on sleep duration were obtained from a question about the average number of hours participants slept per night over the course of the previous month. Based on previous research experience of the CHARLS on sleep [26,27], we divided respondents into seven sleep duration groups (≤4, 5, 6, 7, 8, 9, and ≥10 hours per night) in the analyses. In addition to assessing sleep duration, we also explored daytime napping habits and established four distinct groups based on napping duration: 0, <30, 30-90, and >90 minutes [28].

### Functional Disability

Functional disability was evaluated by asking participants about their ability to perform several domains of routine tasks included in the ADL and IADL questionnaires without special equipment [29]. The response of each item was categorized into four levels: “No, I do not encounter any problem”; “I experience difficulty but am still able to perform the task”; “Yes, I encounter trouble and require assistance”; and “I am unable to perform the task.” According to previous studies [30-32], respondents were recorded as having a functional disability if they reported any challenge in any of the six ADLs (dressing, bathing, eating, getting in and out of bed, using the toilet, and managing urination and defecation) or the five IADLs (household chores, cooking, shopping, paying bills or managing assets, and taking medications) [29,33].

### Covariates

The CHARLS structured questionnaire was completed using in-person interviews to gather participants’ demographic characteristics as covariates, including age (years), gender (man or woman), tobacco use (never and current use), alcohol consumption (never and current use), education level (illiterate, primary or middle school, and high school or higher), residential location (urban or rural area), marital status (married or cohabiting and other statuses), afternoon napping habits, chronic disease status, sampling weights, and depression status. The 10-item Center for Epidemiological Studies Depression Scale was used to differentiate between individuals with and without depression according to a cut-off score of 10 [34-36]. The chronic illness condition of participants was assessed using self-reported noncommunicable diseases (NCDs), including hypertension, diabetes, dyslipidemia, heart issues, stroke, liver diseases, renal diseases, lung diseases, arthritis, and stomach disorders. Based on a recent analysis of CHARLS data on sleep duration [37], individuals were divided into three chronic disease groups: “none” (no NCD), “mild” (1-2 NCDs), or “severe” (more than 3 NCDs). Sampling weights were incorporated based on sets of cross-sectional individual weights that included adjustments for nonresponse by individuals and households.

### Statistical Analysis

Participants’ characteristics are summarized as numbers and percentages for categorical variables and as mean and SD values for continuous variables and were divided according to the functional disability status (including ADL and IADL disability). Missing baseline data were handled by a multiple-imputation method, which is a widely used approach to compensate for missing data via generating predictions for each missing value multiple times, resulting in a data set containing no missing values [38]. Multivariable generalized linear models (GLMs) were established using a binomial family and log-link functions to investigate the associations between sleep duration and functional disability status. Restricted cubic-splines analyses with four specific knots located at the 5th, 25th, 75th, and 95th centiles of the exposure distribution were performed to assess dose-response relationships between sleep duration and functional disability. The GLM and restricted cubic-spline models were adjusted for potential confounders, including age, gender, education, marital status, tobacco and alcohol use, afternoon napping, residential location, depression status, chronic disease condition, and sampling weights. All analyses accounting for the complex survey design of the CHARLS were conducted using Stata version 14.0 (Stata Corp). Statistically significant findings were defined by a two-sided *P* value below .05.

## Results

### Sample Characteristics

A total of 5519 participants (2471 men and 3048 women) were included in this analysis with a mean age of 73.67 years, including 2800 (50.73%) participants with a functional disability, 1978 (35.83%) with an ADL disability, and 2299 (41.66%) with an IADL disability.

Table 1 provides a summary of the baseline characteristics of the participants according to disability status. The average sleep duration was 6.04 hours for the total sample, with the majority of respondents reporting sleep durations less than 7 hours. Moreover, individuals with functional disability exhibited an average reduction in sleep duration in comparison to that of individuals without functional disability (5.80 vs 6.28 hours). A similar pattern emerged among individuals with ADL and IADL disabilities. Notably, older participants; women; nonsmokers; alcohol abstainers; those who are single; rural inhabitants; as well as those with lower educational attainment, depression, and a higher burden of chronic illnesses had greater rates of functional disability.

**Table 1:** Baseline characteristics of participants according to functional disability status.

Characteristics		Total sample (N=5519)	Functional disability			ADL^a^ disability			IADL^b^ disability	
			Yes (n=2800)	No (n=2719)	Yes (n=1978)		No (n=3541)	Yes (n=2299)		No (n=3220)
Sleep duration per night (hours), mean (SD)		6.04 (2.41)	5.80 (2.64)	6.28 (2.12)	5.73 (2.72)		6.21 (2.20)	5.78 (2.71)		6.23 (2.15)
**Hours of sleep per night, n (%)**										
	≤4	1418 (25.69)	900 (32.14)	518 (19.05)	678 (34.28)		740 (20.90)	755 (32.84)		663 (20.59)
	5	792 (14.35)	411 (14.68)	381 (14.01)	273 (13.80)		519 (14.66)	345 (15.01)		447 (13.88)
	6	995 (18.03)	450 (16.07)	545 (20.04)	302 (15.27)		693 (19.57)	364 (15.83)		631 (19.60)
	7	695 (12.59)	259 (9.25)	436 (16.04)	182 (9.20)		513 (14.49)	199 (8.66)		496 (15.40)
	8	885 (16.04)	392 (14.00)	493 (18.13)	268 (13.55)		617 (17.42)	311 (13.53)		574 (17.83)
	9	315 (5.71)	145 (5.18)	170 (6.25)	109 (5.51)		206 (5.82)	111 (4.83)		204 (6.34)
	≥10	419 (7.59)	243 (8.68)	176 (6.47)	166 (8.39)		253 (7.14)	214 (9.31)		205 (6.37)
Age (years), mean (SD)		73.67 (6.41)	74.79 (6.79)	72.53 (5.76)	74.83 (6.87)		73.03 (6.04)	75.16 (6.93)		72.62 (5.78)
**Gender, n (%)**										
	Man	2 471 (44.77)	1099 (39.25)	1372 (50.46)	790 (39.94)		1681 (47.47)	883 (38.41)		1588 (49.32)
	Woman	3048 (55.23)	1701 (60.75)	1347 (49.54)	1188 (60.06)		1860 (52.53)	1416 (61.59)		1632 (50.68)
**Education, n (%)**										
	Illiterate	2067 (37.45)	1230 (43.93)	837 (30.78)	857 (43.33)		1210 (34.17)	1058 (46.02)		1009 (31.34)
	Primary or middle school	3132 (56.75)	1464 (52.29)	1668 (61.35)	1050 (53.08)		2082 (58.80)	1156 (50.28)		1976 (61.37)
	High school or above	320 (5.80)	106 (3.79)	214 (7.87)	71 (3.59)		249 (7.03)	85 (3.70)		235 (7.30)
**Tobacco use^c^, n (%)**										
	Never	3158 (57.93)	1677 (60.61)	1481 (55.18)	1163 (59.58)		1995 (57.02)	1379 (60.62)		1779 (56.01)
	Current	2293 (42.07)	1090 (39.39)	1203 (44.82)	789 (40.42)		1504 (42 98)	896 (39.38)		1397 (43.99)
**Alcohol use, n (%)**										
	Never	3959 (71.73)	2120 (75.71)	1839 (67.64)	1479 (74.77)		2480 (70.04)	1787 (77.73)		2172 (67.45)
	Current	1560 (28.27)	680 (24.29)	880 (32.36)	499 (25.23)		1061 (29.96)	512 (22.27)		1048 (32.55)
Married or cohabiting, n (%)		3754 (68.02)	1780 (63.57)	1974 (72.60)	1248 (63.09)		2506 (70.77)	1434 (62.37)		2320 (72.05)
**Area of residence^d^, n (%)**										
	Rural	4337 (78.65)	2304 (82.34)	2033 (74.85)	1635 (82.70)		2702 (76.39)	1901 (82.72)		2436 (75.75)
	Urban	1177 (21.35)	494 (17.66)	683 (25.15)	342 (17.30)		835 (23.61)	397 (17.28)		780 (24.25)
Depression^e^, n (%)		2131 (38.61)	1312 (46.86)	819 (30.12)	968 (48.94)		1163 (32.84)	1083 (47.11)		1048 (32.55)
**Daytime napping (minutes), n (%)**										
	None	1972 (35.73)	1016 (36.29)	956 (35.16)	713 (36.05)		1259 (35.55)	1576 (39.51)		1126 (34.97)
	≤30	417 (9.22)	244 (8.71)	227 (8.35)	167 (8.44)		304 (8.59)	359 (9.00)		259 (8.04)
	31-90	2058 (37.29)	995 (35.54)	1063 (39.10)	706 (35.69)		1352 (38.18)	1385 (34.72)		1271 (39.47)
	≥90	1018 (18.45)	545 (19.46)	473 (17.40)	392 (19.82)		626 (17.68)	669 (16.77)		564 (17.52)
**Chronic disease condition, n (%)**										
	None	2679 (48.54)	1174 (41.93)	1505 (55.35)	805 (40.70)		1874 (52.92)	945 (41.10)		1734 (53.85)
	Mild	2389 (43.29)	1333 (47.61)	1056 (38.84)	951 (48.08)		1438 (40.61)	1099 (47.80)		1290 (40.06)
	Severe	451 (8.17)	293 (10.46)	158 (5.81)	222 (11.22)		229 (6.47)	255 (11.09)		196 (6.09)

^a^ADL: activity of daily living.

^b^IADL: instrumental activity of daily living.

^c^Missing data for 139 (1.11%) participants.

^d^Missing data for 17 (0.14%) participants.

^e^Defined as a score of 10 or greater on the 10-item Center for Epidemiologic Studies Depression scale.

### Trajectories for Sleep Duration According to Functional Disability Status

**Figure 2:**
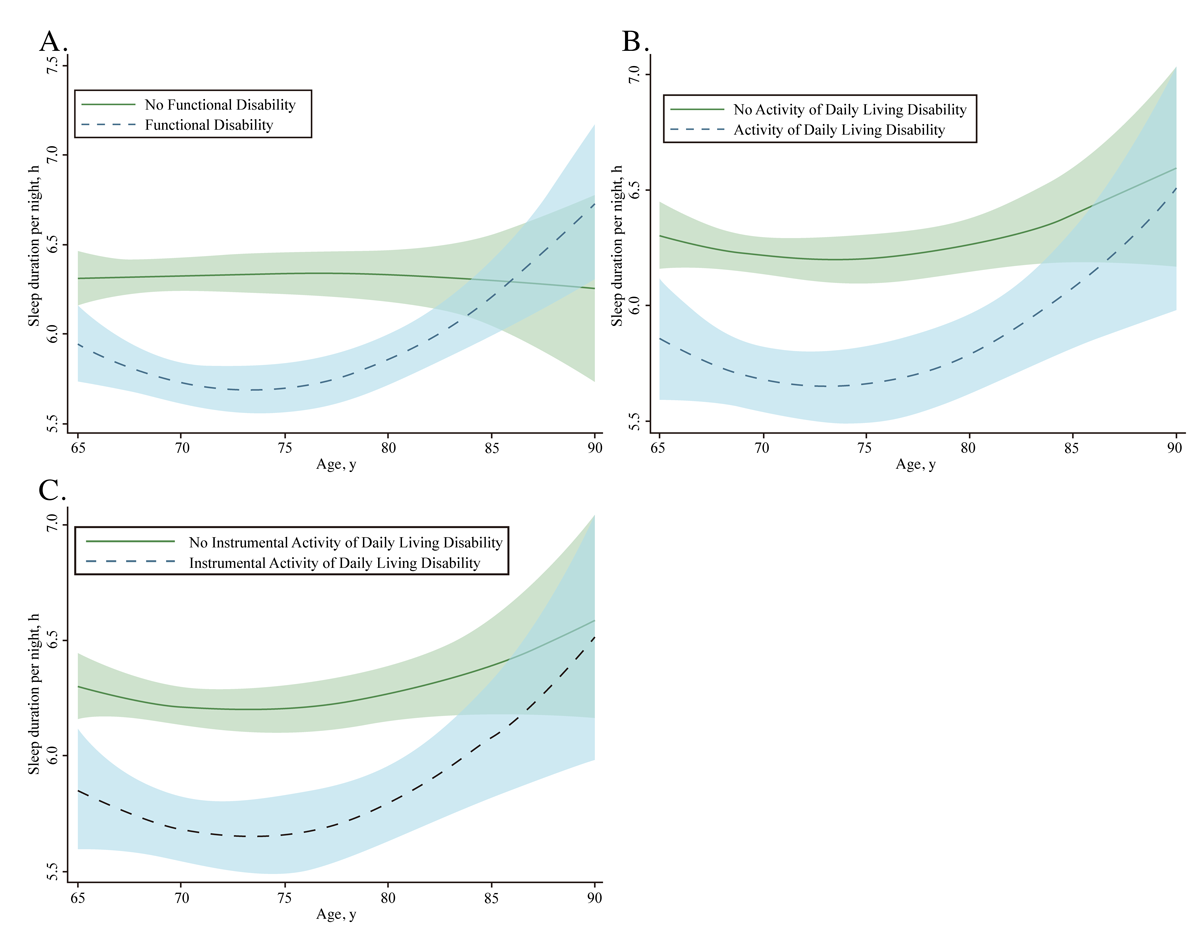
Trajectories of sleep duration across individuals according to functional disability status. Graphs display analog values (lines) of the sleep duration with 95% CIs (shaded areas) for any functional disability (A), activity of daily living disability (B), and instrumental activity of daily living disability (C).

The trajectories of sleep duration for groups classified according to different types of functional disability are depicted in Figure 2. The sleep duration of respondents with no functional disability followed a flat curve, whereas there was a U-shaped association between sleep duration and age among respondents with functional disability (Figure 2A). Individuals in the age range of 65 and 75 years with any functional disability had a substantially shorter sleep duration than those 75 years and older (mean 6.11, SD 2.22 vs mean 5.98, SD 2.43). Respondents with functional disability showed an increasing trend in sleep duration after the age of 75 years (mean change per 1 age point later in life of 0.09 hours). Respondents with ADL disability showed a rapid decline in sleep duration between the ages of 65 and 75 years, which increased after the age of 75 years (Figure 2B). Similar patterns are illustrated in Figure 2C, showing that for respondents with IADL disability, the sleep duration trajectories took on a U shape with age, and the shortest sleep duration was evident around the age of 75 years.

### Associations Between Sleep Duration and Functional Disability

Table 2 presents the association between sleep duration and functional disability status. In the unadjusted model (model 1), both individuals reporting shorter sleep durations (≤4, 5, and 6 hours) and those reporting longer sleep durations (8, 9, and ≥10 hours) per night exhibited a significantly increased risk of functional disability compared to the reference group (7 hours of sleep/night). The same trend was observed in the relationship between different sleep durations and IADL. Correspondingly, participants reporting shorter sleep durations (≤4 and 5 hours) or longer sleep durations (9 and ≥10 hours) demonstrated a significantly higher odds of ADL disability compared to the reference group.

After adjusting for other potential confounding factors (model 2), the significant associations between shorter sleep durations (≤4, 5, and 6 hours) or longer sleep durations (8, 9, and ≥10 hours) and functional disability persisted, whereas the association of a longer sleep duration (9 hours) to IADL disability dissipated and the association of a longer sleep duration (8 hours) to ADL disability emerged (Table 2).

**Table 2:** Associations between sleep duration and functional disability status in participants from the 2018 China Health and Retirement Longitudinal Study.

Sleep duration per night (hours)		Functional disability			ADL^a^ disability			IADL^b^ disability	
		OR^c^ (95% CI)	*P* value	OR (95% CI)		*P* value	OR (95% CI)		*P* value
**Model 1^d^**									
	≤4	2.92 (2.42-3.52)	<.001	2.58 (2.12-3.14)		<.001	2.84 (2.34-3.44)		<.001
	5	1.81 (1.48-2.23)	<.001	1.48 (1.19-1.85)		.001	1.92 (1.55-2.38)		<.001
	6	1.39 (1.14-1.69)	.001	1.23 (0.98-1.52)		.06	1.44 (1.16-1.77)		.001
	7	Reference	—^e^	Reference		—	Reference		—
	8	1.34 (1.09-1.63)	.005	1.22 (0.98-1.52)		.07	1.35 (1.09-1.67)		.006
	9	1.43 (1.09-1.88)	.009	1.49 (1.12-1.98)		.006	1.36 (1.02-1.80)		.04
	≥10	2.32 (1.81-2.97)	<.001	1.85 (1.43-2.39)		<.001	2.60 (2.02-3.34)		<.001
**Model 2^f^**									
	≤4	2.65 (2.08-3.38)	<.001	2.16 (1.68-2.78)		<.001	2.48 (1.94-3.19)		<.001
	5	1.78 (1.34-2.37)	<.001	1.39 (1.04-1.88)		.03	1.82 (1.36-2.46)		<.001
	6	1.67 (1.29-2.15)	<.001	1.26 (0.96-1.66)		.09	1.69 (1.29-2.20)		<.001
	7	Reference	—	Reference		—	Reference		—
	8	1.51 (1.18-1.94)	.001	1.33 (1.02-1.74)		.04	1.48 (1.14-1.93)		.003
	9	1.57 (1.12-2.21)	.01	1.66 (1.16-2.38)		.006	1.37 (0.96-1.96)		.09
	≥10	2.23 (1.60-3.09)	<.001	1.64 (1.17-2.29)		.004	1.91 (1.57-2.32)		<.001

^a^ADL: activity of daily living.

^b^IADL: instrumental activity of daily living.

^c^OR: odds ratio.

^d^Model 1 was unadjusted.

^e^Not applicable.

^f^Model 2 was adjusted for age, gender, education, marital status, tobacco use, alcohol use, afternoon napping, residence, depression, chronic disease status, and sampling weights.

### Subgroup Analyses

**Figure 3:**
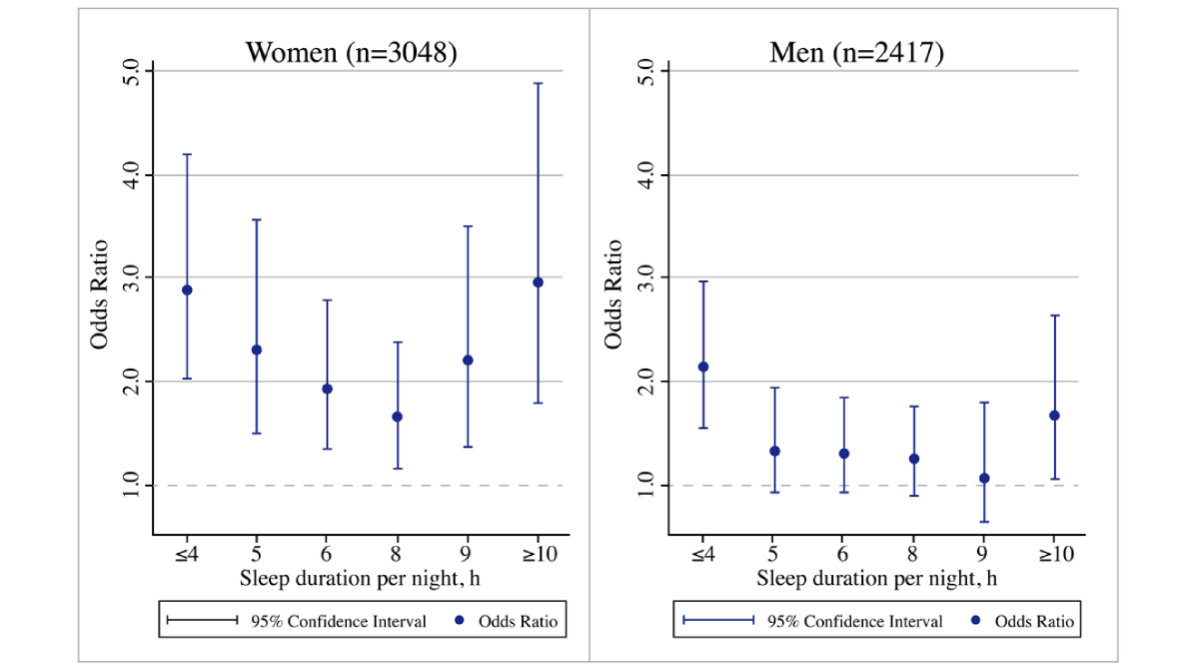
Gender-specific effect of sleep duration on functional disability.

**Figure 4:**
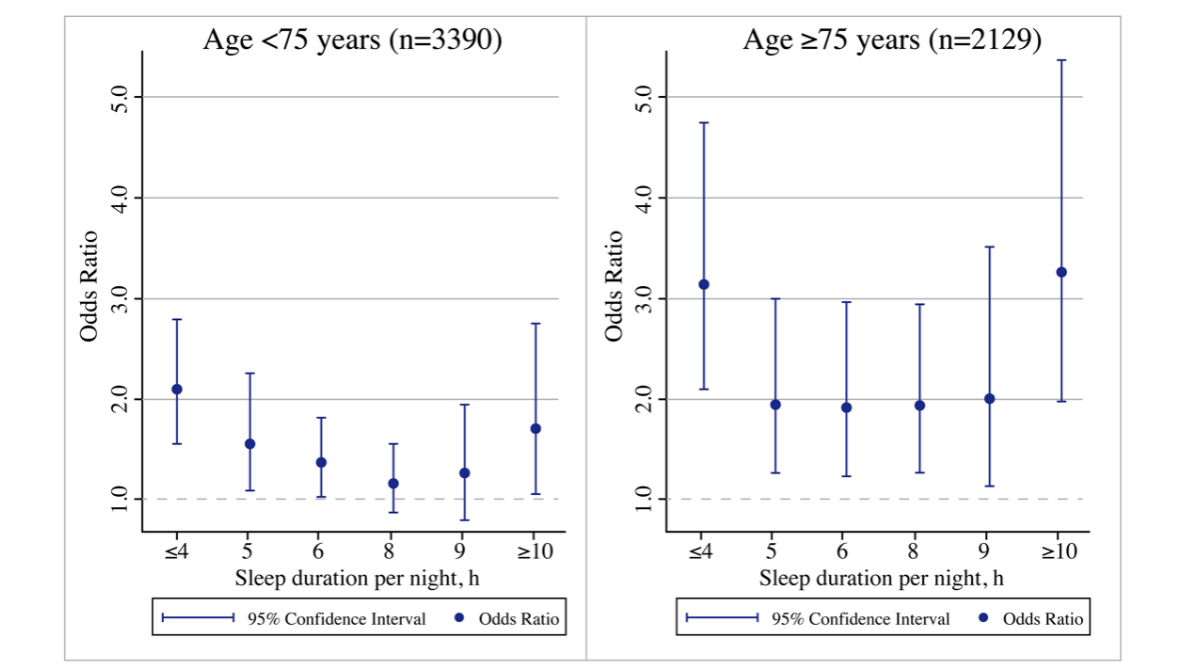
Age-specific effect of sleep duration on functional disability.

The findings of subgroup analyses by gender and age are shown in Figure 3 and Figure 4, respectively, where significant effects specific to gender and age were noted. Compared to men, women who slept for shorter durations (≤4, 5, and 6 hours) or longer durations (8, 9, and ≥10 hours) per night were more likely to experience functional disability (Figure 3). Regarding age-specific effects, participants in the older group (75 years and older) who slept for fewer than 6 hours or more than 8 hours were more likely to develop a functional disability than the younger group (74 years and younger) (Figure 4).

### Nonlinear Relationship Between Sleep Duration and Functional Disability

**Figure 5:**
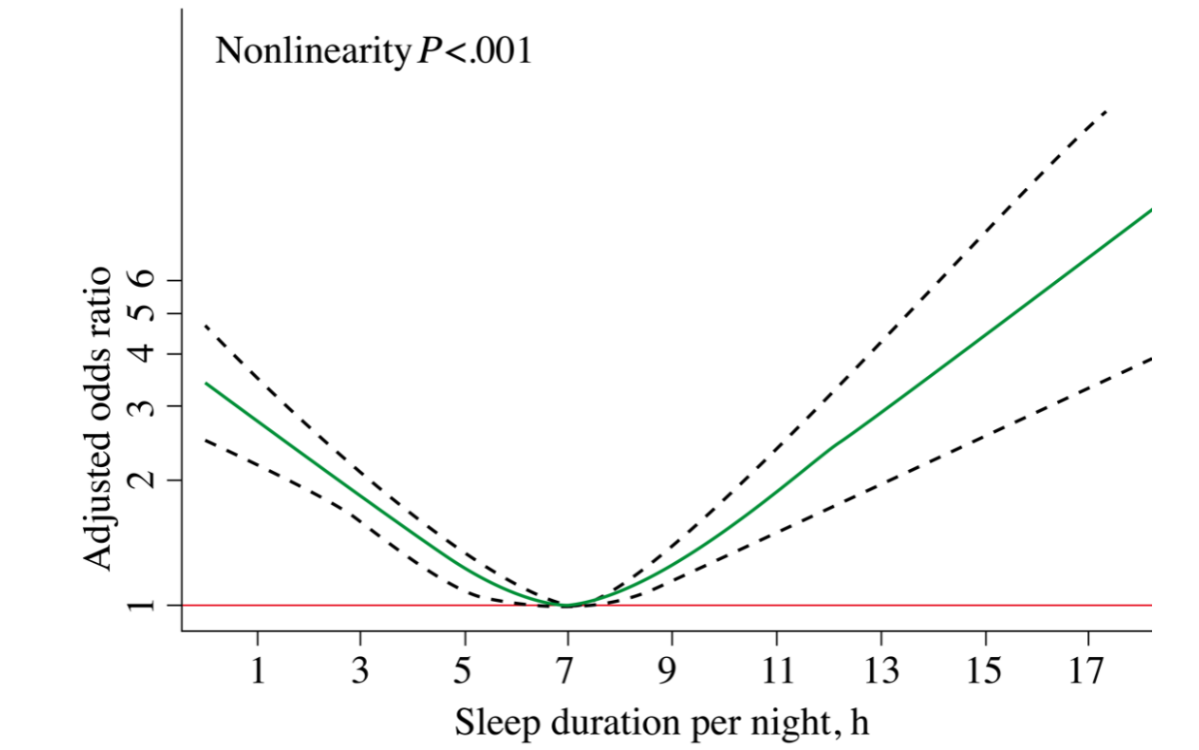
Nonlinear relationship of sleep duration and functional disability status. The adjusted odds ratio is presented accounting for potentially confounding factors (green solid line) in the relationship of sleep duration with 95% CIs (dotted lines) for functional disability. The red solid line is the reference line for the association at an odds ratio of 1.0.

Restricted cubic-splines analyses were conducted to visually represent the associations between the duration of sleep and functional disability. We found a U-shaped relationship between sleep duration and functional disability, even after accounting for confounding factors. As shown in Figure 5, the risk of functional disability was negatively correlated with sleep duration until it bottomed out at 7 hours (odds ratio [OR] 0.85, 95% CI 0.79-0.91; *P*<.001). Nevertheless, there was a substantial increase in the risk of functional disability when the duration of sleep exceeded 7 hours (OR 1.16, 95% CI 1.05-1.29; *P*<.001).

## Discussion

### Principal Results

Our findings obtained from a nationally representative sample of 5519 participants 65 years and older in China indicated that shorter or longer sleep durations are associated with a higher risk of functional disability, including limitations in ADLs and IADLs. In the case of the older population, a minimum risk of 7 hours of sleep is associated with a reduced likelihood of experiencing functional disability. Based on previous studies highlighting functional disability as a notable risk factor for sleep disorders in the older population [39], the findings of this study suggest that the association between sleep and functional disability can exhibit a bidirectional nature.

### Limitations

Our study has several limitations. First, the data of sleep duration and functional disability assessment were collected via self-report by participants, which could be subject to information bias. Second, the sample for this study comprised individuals from China who were 65 years of age or older. This particular demographic characteristic may limit the generalizability of the findings to different age cohorts, geographical areas, or ethnic backgrounds. Third, this study adopted a cross-sectional design, which limits the ability to show a causal association. It is plausible to consider that older adults with functional disability may require prolonged periods of sleep and a reduced duration of sleep could potentially serve as an initial indication of dysfunction. Therefore, it is necessary to conduct further comprehensive cohort studies to validate these findings.

### Comparison With Prior Work

Functional ability refers to an individual’s capacity to engage in daily living and social activities according to their own intentions and preference. However, it is discouraging that the prevalence of functional disability was determined to be 41% across the entire sample in China [40]. The aging process is commonly accompanied by disturbances in sleep patterns, which have been linked to notable alterations in brain function and a decline in overall quality of life [41]. Moreover, various population-based studies [42-44], including both cross-sectional and longitudinal study designs, have demonstrated that the prevalence of cognitive decline may be linked to an increased risk of experiencing functional disability. Epidemiological research has also documented a U-shaped association between sleep duration and cognitive decline, indicating a significant trend in the association between sleep duration and functional disability [45-47]. Nevertheless, a definitive consensus has not yet been attained and there is still a dearth of studies examining the association between sleep duration and functional disability.

Despite previous investigations into the association between sleep duration and self-care function, the majority of these studies have primarily concentrated on a single form of functional disability. One study involving nightshift workers in the United States found that individuals with a shorter sleep duration (<7 hours/day) had the highest prevalence of sleep problems (61.8%) and the highest prevalence of an impaired ADL score (24.8%) [48], revealing a correlation between shorter sleep duration and higher risk of ADL disability. Another study focusing on patients with dementia discovered that a longer sleep duration was associated with ADL disability [49]. Similarly, a cross-sectional study that recruited 1798 participants older than 90 years found that a long sleep duration (≥12 hours) may be associated with an increased risk of ADL disability for this population [19]. Peng et al [50] found that after accounting for potential confounders such as age and gender, both longer and shorter sleep durations were linked to a heightened risk of IADL disability, which was consistent with the results of this study. Furthermore, instead of solely focusing on the relationship between shorter or longer sleep duration and functional disability among different sample sets, we simultaneously explored the impact of both shorter and longer sleep durations on functional disability within the same sample group. Additionally, concerning functional disability, we separately explored the associations between sleep duration and ADL and IADL disabilities, demonstrating that both shorter and longer sleep durations have an influence on functional health among the older population.

### Implications and Contribution

The mechanism that accounts for the association between sleep duration and functional disability has yet to be fully explained; however, several hypotheses have been proposed. Luo et al [51] demonstrated that inadequate and excessive sleep durations were associated with an increased likelihood of hypertension among Chinese individuals. The increased susceptibility to cardiovascular illnesses associated with this elevated risk can have a direct influence on the overall well-being and functional abilities of older adults. Another recent study revealed that both insufficient and excessive sleep durations are associated with an increased risk of late-life dementia, a condition that significantly impairs the ability of older individuals to perform their daily tasks [52]. Other relevant studies indicated that sleep was correlated with changes in epigenetic mechanisms such as DNA methylation and histone modifications, which can also lead to cognitive dysfunctions such as learning and memory disruption [53-55]. This evidence has provided a new avenue for exploring the mechanism underlying the relationship between sleep duration and functional disability. Interventions aimed at good sleep hygiene may have the capacity to yield favorable outcomes in terms of improving physiological function among older adults. As evidenced by the empirical findings of this study, maintenance of the recommended sleep duration (7 hours) might play a crucial role in the health of older adults.

Our subgroup analyses suggested that women and older adults aged ≥75 years with shorter sleep durations (≤4, 5, and 6 hours) or longer sleep durations (8, 9, and ≥10 hours) per night were more likely to experience functional disability. Gender differences in sleep duration among the older population cannot be ignored. With changes in biological life cycles and the extreme hormonal fluctuations occurring with advancing age, women are at an increased risk for sleep disturbances (including insomnia and hypersomnia) [56]. Further, the sleep disturbances occurring during menopause can be an independent risk factor associated with arterial stiffness, which can result in a higher incidence of osteoarthritis that is in turn highly related to dysfunction [57]. With respect to age-specific effects, changes in sleep duration are a part of the normal aging process and also may enhance cellular aging in the later years of life [58]. According to the findings from an umbrella review [59], extreme sleep durations (including shorter and longer sleep durations) were more likely to be associated with an elevated risk of all-cause mortality, cognitive disorders, and type 2 diabetes in the general population. The circadian oscillations that alter body functions, including sleep, become less pronounced in older age, which can increase the risk of functional disability [60]. Collectively, these results emphasize the importance of addressing the complex needs of the population experiencing functional disabilities, particularly among women and older adults.

### Conclusions

In conclusion, more attention should be paid to older individuals with shorter or longer sleep durations than recommended (7 hours). The precise mechanisms underlying the association between sleep duration and functional disability in the older population require further investigation.

## Abbreviations

ADL: activity of daily living
CHARLS: China Health and Retirement Longitudinal Study
GLM: generalized linear model
IADL: instrumental activity of daily living
NCD: noncommunicable disease
OR: odds ratio
STROBE: Strengthening the Reporting of Observational Studies in Epidemiology
WHO: World Health Organization

## Data Availability

The data sets presented in this study can be found in online repositories of the China Health and Retirement Longitudinal Study [61]. The data sets generated and analyzed during this study are available from the corresponding author on reasonable request.
